# Species-level bacterial community shift in rice growth stages

**DOI:** 10.5511/plantbiotechnology.25.0310b

**Published:** 2025-09-25

**Authors:** Sachiko Masuda, Kazuhiro Sasaki, Arisa Shibata, Tadashi Sato, Ken Shirasu

**Affiliations:** 1 RIKEN Center for Sustainable Resource Science, RIKEN-TRIP, Kanagawa 230-0045, Japan; 2 Institute for Sustainable Agro-ecosystem Services, Graduate School of Agricultural and Life Sciences, The University of Tokyo, Tokyo 188-0001, Japan; 3 Graduate School of Life Sciences, Tohoku University, Miyagi 980-8577, Japan; 4 Graduate School of Science, The University of Tokyo, Tokyo 113-0033, Japan

**Keywords:** bacterial community, rice-phyllosphere, 16S rRNA full-length amplicons

## Abstract

The phyllosphere is a major microbial habitat, where resident communities promote plant growth, suppress pathogens, and induce disease resistance. Here we examined how rice growth stages influence microbial colonization by analyzing bacterial communities in the phyllospheres of three growth stages (panicle initiation, heading, harvesting) across three genotypes: ‘Koshihikari’ and two introgression lines. Bacterial communities were similar among genotypes in both leaves and stems at heading but became distinct at harvesting, indicating that growth stages and plant organ play primary roles in shaping community structure. Full-length 16S rRNA gene amplicon sequencing further revealed significant shifts in species composition, with *Pseudomonas* species, such as *Pseudomonas brenneri* and *Pseudomonas helmanticensis*, were consistently present across organs and stages, while *Enterobacter* species showed stage-specific colonization. These findings highlight the dynamic nature of phyllosphere microbial communities throughout plant development and underscore the importance of organ- and stage-specific factors in shaping plant-microbe interactions.

## Introduction

The phyllosphere, aerial parts of plants, represents the largest environmental interface for microbial colonization on Earth (Sohrabi et al. 2023). Microbes inhabiting this niche can directly enhance plant growth by improving nutrient uptake, suppressing pathogens, and inducing disease resistance (Trivedi et al. 2020). The community assembly arise from dynamic interplay between host and environment: the plant exerts selective pressure via its immune system and exudates (Ryffel et al. 2016; Vogel et al. 2016), while environmental factors such as UV and temperature also play important roles in shaping phyllosphere microbiome (Aydogan et al. 2018; Jacobs and Sundin 2001). Consequently, phyllosphere communities are co-determined by both biotic and abiotic factors (Li et al. 2021). Furthermore, plant developmental stages modulate those microbial assemblages in concert with season-dependent environmental factors (Xiong et al. 2021). Previously, we reported that shoot bacterial communities in rice are significantly influenced by plant genotype, whereas root microbiomes are largely affected by the nitrogen fertilization level (Sasaki et al. 2010). However, because plant growth stage was not evaluated in the study, the possibility remains that the developmental stages also drive bacterial colonization in rice, leaving open the question of which specific factors govern bacterial community assembly in this crop.

Proteobacteria, Bacteroidetes, Firmicutes, and Actinomycetes, have been widely recognized as the dominant phyla in the plant phyllosphere, as evidenced by both culture-dependent and culture-independent methods (Vorholt et al. 2017). Although these approaches provide valuable insights into microbial diversity and functional potential, each is constrained by inherent limitations. Cultivation-based methods offer only a limited perspective, as many microorganisms remain unculturable in vitro. Similarly, metagenomic amplicon sequencing of the 16S rRNA gene using short reads often lacks the resolution needed to distinguish bacteria at the species level (Masuda et al. 2024). Consequently, our understanding of which bacterial species colonize the rice phyllosphere remains incomplete.

Our previous study addressed these classic challenges by employing long-read metagenomics, uncovering novel bacterial species, genes, and plasmids in the rice phyllosphere microbiome (Masuda et al. 2024). Remarkably, three-quarters of the 16S rRNA genes detected in the study were inferred to represent novel species. While long-read metagenomics significantly enhances our understanding of the bacterial community composition within the rice phyllosphere, our findings so far capture only a snapshot of its community structure. It remains unclear which bacterial species dominate specific parts of the rice-phyllosphere and whether their colonization patterns persist across different developmental stages.

Here, we performed bacterial community analysis in rice phyllosphere to answer two questions: 1) which specific factors govern bacterial community assembly in rice phyllosphere, 2) which bacterial species colonized in the rice phyllosphere across different developmental stages. To address the first question, we cultivated one rice cultivar (‘Koshihikari’) and two introgression lines (SL526 and SL527) in an experimental field to investigate the composition of the rice-phyllosphere microbiome. We collected leaves, stems, and panicles at three growth stages: panicle initiation (PI), heading, and harvesting. The two lines, derived from a cross of a *japonica* cultivar, Koshihikari and an *indica* cultivar, ‘Nona Bokra’, exhibit late heading under long-day conditions (Takai et al. 2007). Preliminary investigations have shown that the SL526 and SL527 take approximately four and two weeks longer, respectively, to start heading compared to Koshihikari because of introgressed segment from Nona Bokra. Using these genotypes, for example, even if the stage of heading is the same, the environment, such as temperature, humidity and rainfall, should be different. Thus, we hypothesized that if developmental stage were the primary determinant of community composition, bacterial profiles would differ by growth stage rather than genotype. Conversely, if environmental factors are paramount, bacterial profiles would vary with genotype. For answer the second question, we identified bacterial species using 16S rRNA full-length amplicon sequences across the three growth stages to elucidate colonization patterns throughout rice development.

## Materials and methods

### Sampling and DNA extraction

Rice plants (*Oryza sativa* cultivar ‘Koshihikari’, ‘SL526’ and ‘SL527’) were grown in an experimental paddy field at the Institute for Sustainable Agro-ecosystem Services, Graduate School of Agricultural and Life Sciences, The University of Tokyo (35°74′N, 139°54′E). Plant samples (*n*=4) were collected at three growth stages: PI, heading and harvesting. At PI stage, leaves and stems were collected. At the heading and harvesting stages, leaves and stems were collected, while panicles were sampled separately. Tissues were ground with mortar and pestles, and microbes were purified through cell density centrifugation (Ikeda et al. 2009; Masuda et al. 2024). Genomic DNA was then extracted as described previously (Masuda et al. 2024).

### Sequencing of phyllosphere microbiome

The V4 region of 16S rRNA genes from leaves, stems, and panicles of the three cultivars across the three growth stages was amplified using the primers 515F (5′-ACA CTC TTT CCC TAC ACG ACG CTC TTC CGA TCT GTG CCA GCM GCC GCGGTA A-3′) and 806R (5′-GTG ACT GGA GTT CAG ACG TGT GCT CTT CCG ATC TGG ACT ACH VGG GTW TCT AAT-3′). And sequenced using the Illumina MiSeq v3 platform. Additionally, full-length 16S rRNA genes from Koshihikari samples collected at three growth stages were amplified following a previously described method (Masuda et al. 2024), sequenced on a SMRT Cell 8M platform, and processed to generate HiFi reads using SMRT Link v8.0.

### Data analysis

The non-metric multidimensional scaling (NMDS) based on Bray–Curtis distance using permutational multivariate analysis of variance using distance matrices (Adonis test) from the Vegan and visualized using ggplot2 in R (https://www.R-project.org/). The DADA2 v1.16.10 (Callahan et al. 2016) was used to denoise and remove the sequencing error reads from 16S rRNA full-length datasets as previously described (Masuda et al. 2024). To analyze the bacterial community composition at the species level, reads with >97% identity to reference strains were extracted (Masuda et al. 2024). The relative abundance was compared between samples, using Kruskal–Wallis for Miseq data and ANOVA for 16S rRNA full-length amplicons. Co-occurrence network analysis was calculated by network (threshold 0.7) (Hagberg et al. 2008).

### Data availability

The Miseq v4 region and PacBio 16S rRNA full-length sequencing data have been deposited in NCBI under accession number PRJNA1202664 (BioProject).

## Results and discussion

### Growth stage and plant organs exert a marked influence on the rice phyllosphere microbiome

**Figure 1:**
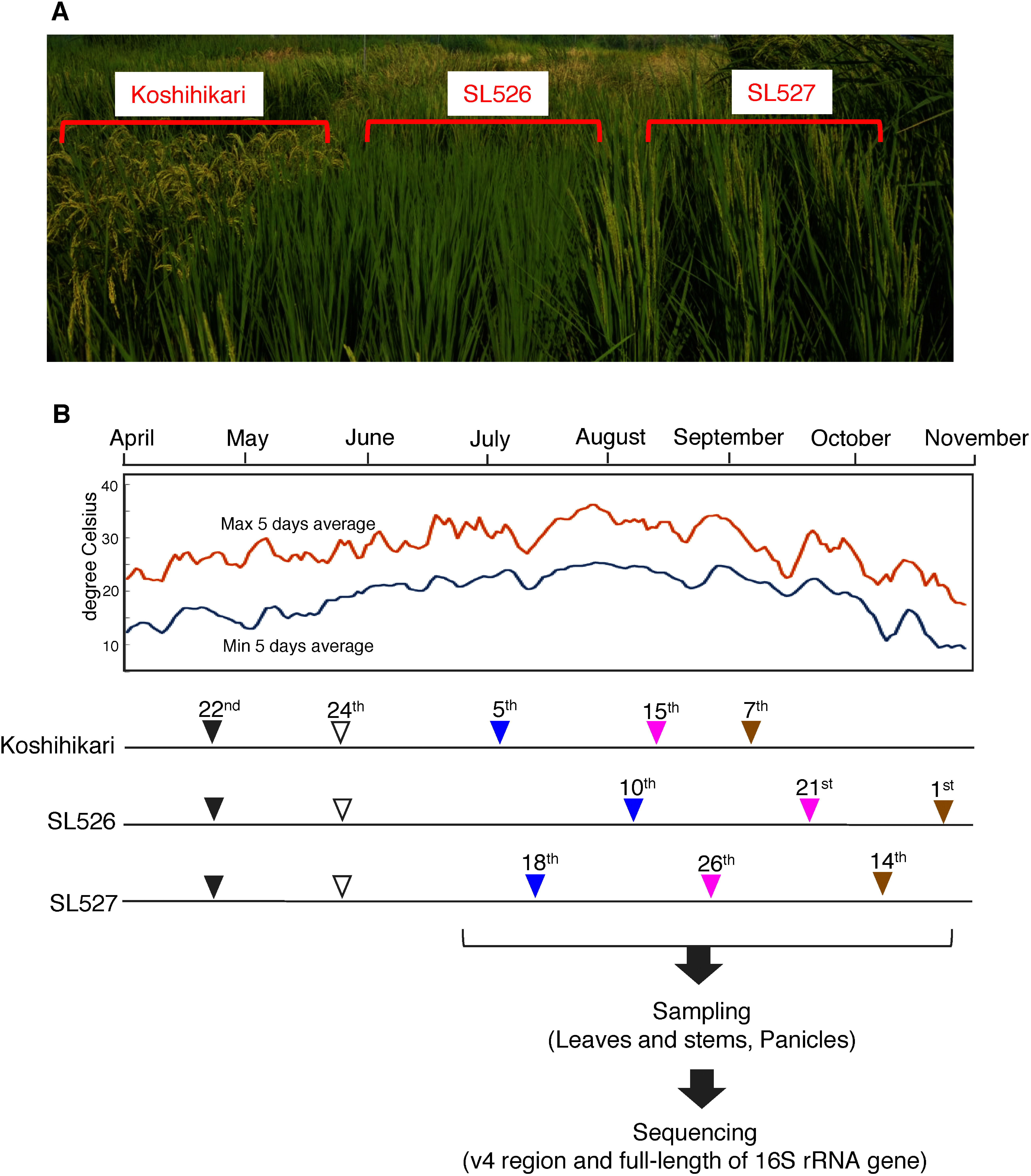
Figure 1. Three rice cultivars growing in the experimental field. A, The picture was taken at the harvesting stage (9th of September) of Koshihikari. B, Five-day average maximum and minimum temperatures in the field throughout the rice growing season, along with the cultivation schedule and sampling date for each cultivar marked by downward triangles (dark grey, seeding; white, transplantation; blue, PI; pink, heading; yellow, harvesting). Above-ground plant tissues (leaves and stems, and panicles) were collected and subjected to Miseq-based v4 16S rRNA gene sequencing. Full-length 16S rRNA genes for Koshihikari samples were additionally sequenced on PacBio Sequel II.

**Figure 2:**
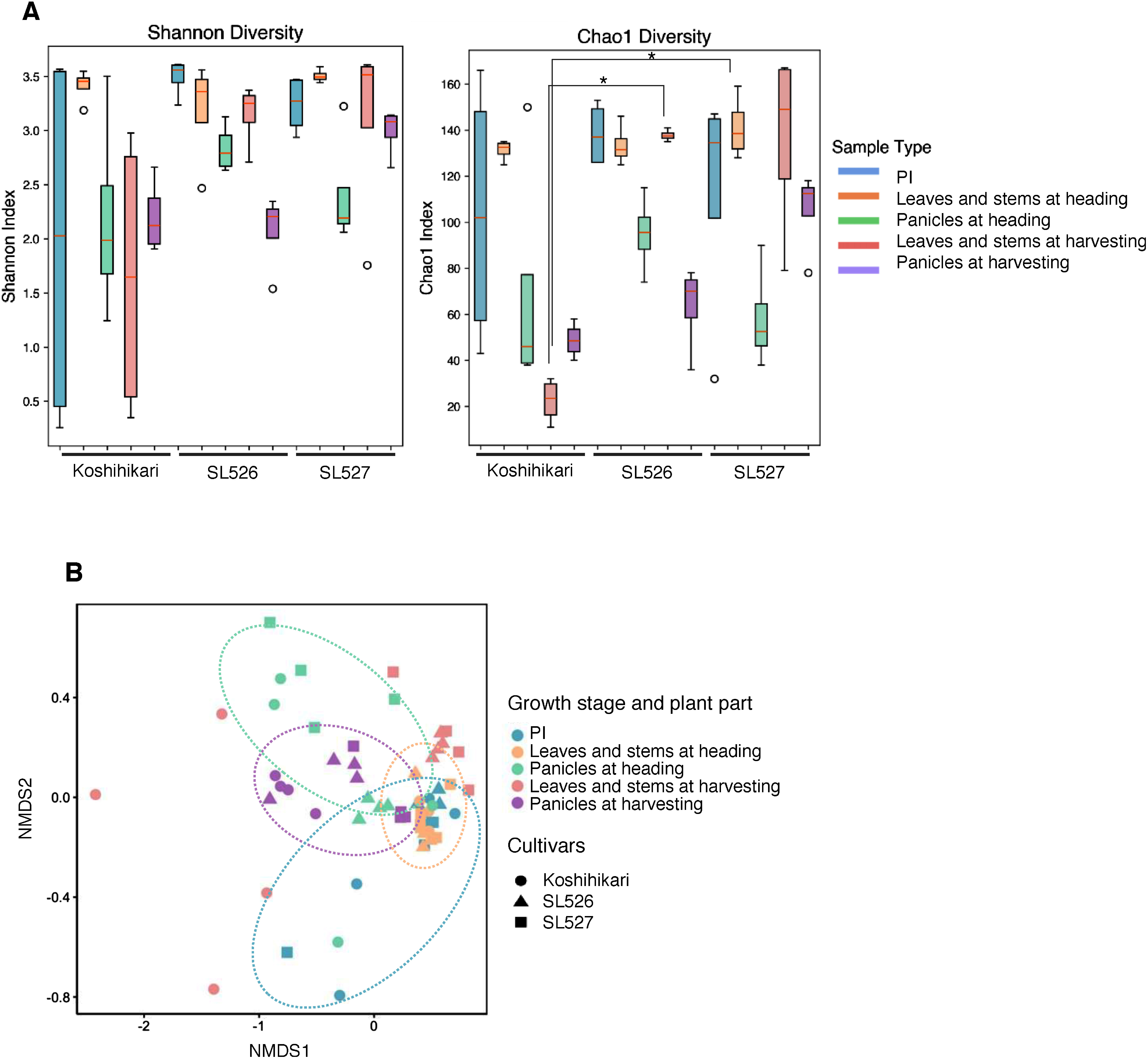
Figure 2. NMDS plots showing the bacterial community composition of different plant parts across three growth stages in three rice cultivars. A, On the left panel, boxplots show Shannon index diversity; on the right, Chao index richness. Within each box, the lower, middle, and upper horizontal lines in the boxes correspond to the first, second, and third quartiles (the 25th, 50th, and 75th percentiles). The vertical extending lines denote adjacent values. The dots denote observations outside the range of adjacent values. * Stands for *p*-value<0.05. B, Each plot represents a sample (*n*=4), indicating cultivar, growth stage and plant part. The grouping was based on the PERMANOVA (<0.001).

To investigate factors influencing the bacterial community on the rice-phyllosphere, we collected leaves, stems, and panicles from three rice genotypes at three developmental stages, as described in Figure 1. The bacterial community composition was assessed by sequencing the V4 region of 16S rRNA genes and compared using Non-Metric Multidimensional Scaling (NMDS) analysis, with each plot representing individual rice samples (*n*=4, Figure 2). The NMDS analysis revealed that the bacterial communities on leaves and stems at the heading stage and on the panicles at harvesting were closely related across all three genotypes (PERMANOVA <0.001), indicating that bacterial composition was largely consistent regardless of genotype. However, these communities clustered separately by plant organ, suggesting that rice parts, rather than genotype, play the primary role in structuring the rice phyllosphere microbiome. In contrast, at the harvesting stage, the bacterial communities on leaves and stems were distinct among the three genotypes. This observation might reflect variations in plant growth and metabolic states, as some leaves remained alive while others were senescing. Senescing leaves often harbor an increased relative abundance of α-Proteobacteria and enhanced microbial diversity (Lan et al. 2024). It is also known that the bacterial community composition differ between the PI and ripening stage (Okubo et al. 2014). Since the carbon and nitrogen metabolism are changed during the rice life cycle (Kim and Sung 2023; Yang et al. 2022), the microbial community changed due to variations in available nutrients. Consequently, coexistence of leaves at different developmental likely contributed to the distinct microbial profiles observed in each plant at harvesting. Although significant differences were not observed, the Shannon’s diversity of the bacterial community looked higher in PI and leaves and stems at heading stage across three cultivars, and lower in the panicles at the harvesting stage across three cultivars (Figure 1). In addition, the Shannon’s diversity index looked lower in the panicles at heading and harvesting stage. The significant differences (*p*<0.05) were observed in Chao1 index (leaves and stems at harvesting stage of Koshihikari and SL526, and leaves and stems at harvesting stage of Koshihikari and leaves and stems at heading stage of SL527).

In our field experiment, uncontrolled variables made it challenging to pinpoint the specific factors affecting bacterial community composition. Nevertheless, both biotic and abiotic conditions likely contributed to the observed shifts. Given that the three rice genotypes exhibited different heading dates (Figure 1), our results suggest that rice growth stage and plant organ, rather than environmental factors, were the primary drivers of the bacterial community composition. Thus, the early growth stage of the rice-phyllosphere provides an ideal window for studying the factors influence bacterial assemblages.

### Identification of bacterial species transition in rice

**Figure 3:**
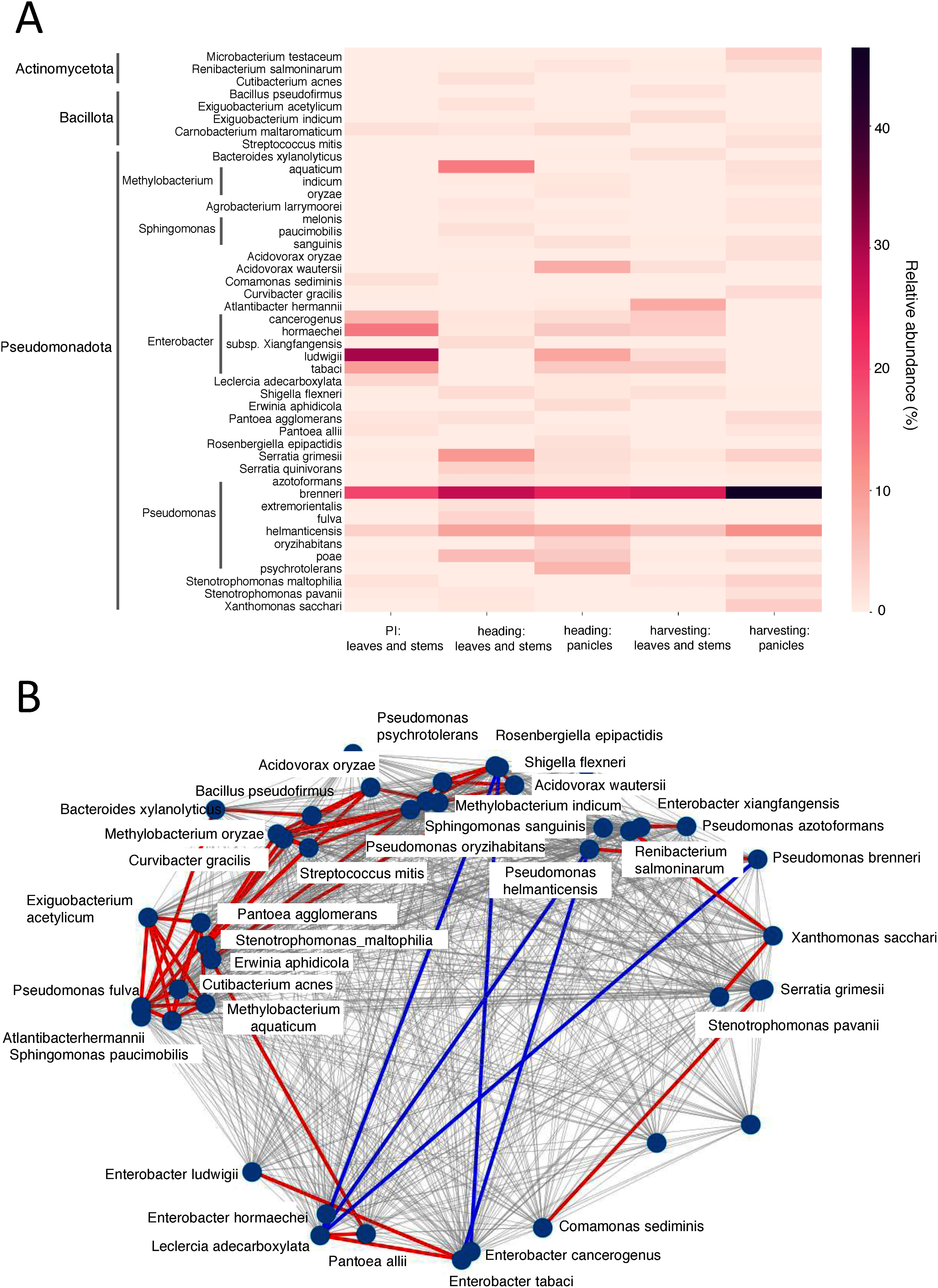
Figure 3. The colonization pattern of the species. A, Relative abundance of the species detected at greater than 1% even in leaves, stems and panicles across three growth stages. B, Co-occurrence network analysis, where red and blue lines indicate positive and competitive correlations, respectively. Each plot represents the bacterial species detected in this study.

**Figure 4:**
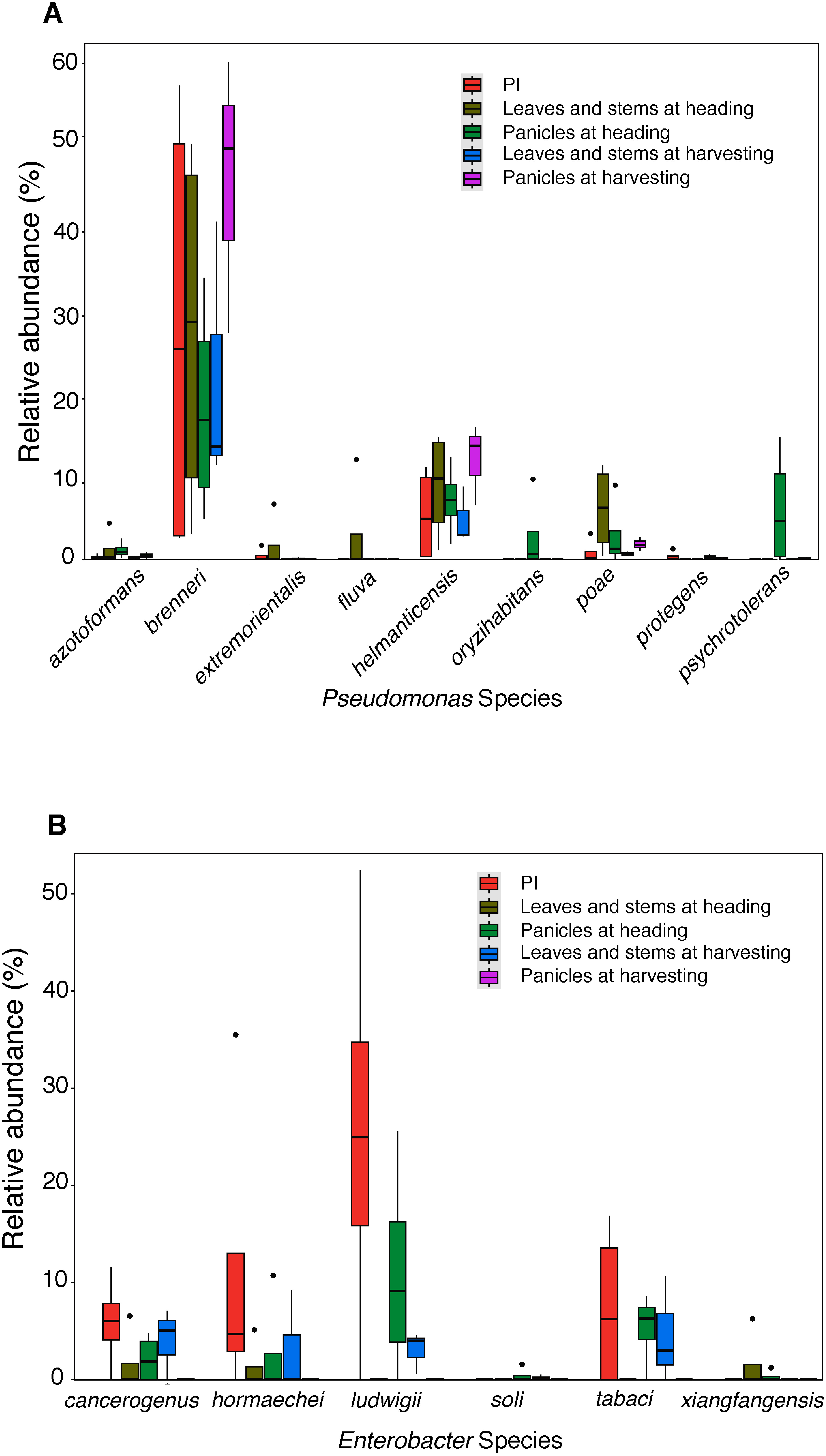
Figure 4. The colonization pattern of *Pseudomonas* and *Enterobacter* species. The colonization pattern of *Pseudomonas* (A) and *Enterobacter* (B) species in rice parts of three developmental stages with their relative abundances. Within each box, the horizontal black line represents the median value. The box extends from the 25th to the 75th percentile of each group’s distribution of values, while the vertical lines indicate the adjacent values. Dots represent observations outside the range of adjacent values.

Next, we sequenced the full-length of 16S rRNA genes to identify the bacterial species colonizing different growth stages and plant organs in cultivar Koshihikari because of long-read sequencing provide more accurate identification about bacterial communities (Masuda et al. 2024). Finally, we extracted 273,588 16S rRNA sequences and clustered into 83 species with 97% identity as previously described (Masuda et al. 2024). The 70 were ≥97% identical to sequences of known taxa, but 6 and 5 showed ≤97% and ≤94.5% identity, suggesting that they potentially represent a novel genus and family, respectively (Table 1). Among latter, we obtained potentially one novel class and order (Table 1). These results suggested that we clarified potentially novel taxonomy using 16S rRNA full-length amplicons. The 45 clusters had relative abundances greater than 1% in leaves, stems or panicles across the three growth stages (Figure 3A). Notably, some species showed site specific increases in relative abundance, and the even closely related species within the same genus exhibited distinct colonization patterns. For example, *Methylobacterium aquaticum* and *Serratia grimesii* were abundant during the heading stage, whereas other members of these genera were not. Similarly, *Acidovorax wautersii* was abundant in panicles at heading, while *Acidovorax oryzae* was undetected. Co-occurrence network analysis revealed positive correlations (red lines), indicating similar colonization patterns, and negative correlations (blue lines), reflecting competition among community members (Figure 3B). These results suggested that colonization dynamics were not strictly determined by taxonomic identity. Some species from different genera exhibit similar colonization patterns, whereas species within the same genus did not necessarily behave alike (Figure 3A, B). For example, in *Pseudomonas* species, *P. helmanticensis* and *P. brenneri* were co-occurred, while the other *Pseudomonas* species did not exhibit the same behavior as *P. helmanticensis* and *P. brennerii*. The *Pseudomonas* species except for *P. helmanticensis* and *P. brennerii* were not positively or negatively correlated to each other. However, there was competition among *P. helmanticensis* and *Enterobacter tabaci*, or *Enterobacter hormaechei* (Figure 3B). Both *Pseudomonas* and *Enterobacter* species were particularly abundant, suggesting that their behavior could significantly influence plant growth. Indeed, two *Pseudomonas* species known for their plant-growth-promoting capabilities, *P. brenneri* (Ferchichi et al. 2019) and *P. helmanticensis* (Ramírez-Bahena et al. 2014), were found in high abundance across all three developmental stages (*P. brenneri*, 19.3–46.4%; *P. helmanticensis*, 3.6–11.3% shown in Figure 4A). These species were also detected in panicles at both heading and harvesting, with their abundance appearing to increase at harvesting, although not significantly. These results indicate that both species initially colonized leaves and stems, then spread to and successfully inhabited the panicles, which aligns with previous reports of their presence in rice seeds (Kim et al. 2020; Mano and Morisaki 2008). Interestingly, other species, such as *Pseudomonas extremorientalis*, *Pseudomonas fulva* and *Pseudomonas poae*, were more abundant in leaves and stems at heading, while *Pseudomonas oryzihabitans* and *Pseudomonas psychrotolerans* were detected only in panicles at that stage.

**Table 1:** Table 1. 16S rRNA genes above the threshold for bacterial taxonomy detected in this study.

Identity (%)	Taxonomic rank	Number of 16S rRNA genes	Number of the clusters
97	species	266,526	70
94.5	genus	1,665	6
86.5	family	5,169	5
82	order	273	1
78	class	38	1

Members of the *Enterobacteriaceae* family play pivotal roles in promoting plant growth through nitrogen fixation, pathogen suppression, and hormone production (Khalifa et al. 2016; Lin et al. 2012; Sallam et al. 2024; Taghavi et al. 2009). In this study, five species of *Enterobacter* exceeded 1% relative abundance in leaves, stems, or panicles across the three growth stages. Four of these species (*Enterobacter cancerogenus*, *Enterobacter hormaechei*, *Enterobacter ludwigii*, and *Enterobacter tabaci*) exhibited high abundance (6.7–30%) during the PI stage, with *E. ludwigii* emerging as the dominant species (Figure 4B). Notably, these species shared similar colonization patterns. For example, the relative abundances of *E. ludwigii* and *E. tabaci* were minimal (0.0–2.1%) in leaves and stems at heading but increased in panicles (2.1–8.6%) at the same stage. However, neither species was detected in panicles during harvesting. Similarly, *E. cancerogenus* and *E. hormaechei* were observed in leaves, stems, and panicles at heading but were absent in panicles at harvesting.

Our findings demonstrate that bacterial colonization in the rice-phyllosphere differ in both timing and location among different species, suggesting that their distribution is selectively influenced by growth stage and plant organ. The bacterial community is influenced by the plant tissue depend on the growth stage. Maize microbiome assembly is mainly influenced by plant part and developmental stage regardless of farming regions and fertilization regimes, and *Actinobacteria* in maize-phyllosphere is more abundant at the seedling stage than at the tasseling stage and the mature stage (Xiong et al. 2021). Plant seasonal status significantly affects microbiomes in the grass-phyllosphere, with Gammaproteobacteria increasing during early growth stages, while Alphaproteobacteria become more dominant in later stages (Grady et al. 2019). Our study suggested that *Pseudomonas* and *Enterobacter* in Gammaproteobcteria was increased at several organs at the different growth stage (Figure 3A, Figure 4A, B). Both *Pseudomonas* and *Enterobacter* species appear to play important and stage-specific roles in rice development. For instance, *P. brenneri* and *P. helmanticensis* were found in leaves, stems, and panicles, implying their potential contributions to plant growth, such as through phosphate solubilization (Lehtola et al. 1999; Ramírez-Bahena et al. 2014). Since phosphorus content in rice seeds has direct effect on seedling vigor and crop establishment as it affects root development and associated physiological processes (Yugandhar et al. 2022), their spread from leaves to panicles underscores a broader role in supporting rice growth. Conversely, *Enterobacter* species like *E. ludwigii* and *E. tabaci* were abundant at the PI stage but declined or disappeared by heading and harvesting. Such community transitions have been observed on the phyllosphere of crops such as sugarcane, common beans, soybeans, and canola (Grady et al. 2019). It remains unclear what drives the community transitions, but a possibility was explained by plant development, which regulates nutrient availability (Delmotte et al. 2009). The colonization in the seeds of *Enterobacter* is limited by reduced carbon compounds, and variations in carbon source utilization impact *Enterobacter* colonization depending on the plant species and seed exudates (Roberts et al. 1999). These results reflect the dynamic nature of plant-microbe interactions across developmental stages. However, 16S rRNA full-length amplicon sequencing provided more comprehensive taxonomic assignments. The full-length of 16S rRNA genes provided detailed insights at the species level, enabling identification of the individual strains within these taxonomic ranks in the rice-phyllosphere.

Our previous study identified *Curtobacterium*, a member in *Actinobacteria*, as the dominant species in the rice phyllosphere (Masuda et al. 2024). However, in the present work, *Curtobacterium* was not detected at high relative abundance. This discrepancy may be attributed to differing fertilizer regimes: while our earlier study utilized organic fertilizer, but the current study employed chemical fertilizer. Several studies have reported that bacterial community composition is influenced by organic and chemical fertilizers, which alter soil pH, carbon, and nitrogen levels (Dai et al. 2018; Ren et al. 2020; Sun et al. 2015). *Actinobacteria*, in particular, is more abundant in agricultural soils with increased organic fertilizer and reduced chemical fertilizer compared to conventional chemical fertilization or unfertilized conditions (Wu et al. 2020). The growth of *Actinobacteria* has been linked to nitrogen input, as organic carbon in the soil significantly increases with nitrogen fertilization. Since most *Actinobacteria* are copiotrophic, they thrive in agricultural soil with low carbon use efficiency (Dai et al. 2018). The low abundance of *Curtobacterium* in this study may be due to the fast nutrient release of chemical fertilizers, which accelerates nutrient consumption by *Curtobacterium* as well. Given its fast growth and high carbon use efficiency, *Curtobacterium* may be less competitive under chemical fertilizer conditions. Consequently, these results suggested the phyllosphere microbiome may shift in response to variations in experimental conditions or developmental stages.

In conclusion, growth stage and plant organs emerged as key factors in shaping microbiome in the rice-phyllosphere. Our study revealed that microbial composition and colonization patterns vary significantly across different growth stages and tissues, highlighting the importance of understanding microbial community dynamics in the context of plant development. Such insights are crucial for optimizing plant health and growth through targeted microbial management strategies. Further studies should delve into the molecular mechanisms that governs host-microbe interactions and drive successful colonization throughout rice growth.
